# Structural and Functional Asymmetry in Precentral and Postcentral Gyrus in Patients With Unilateral Chronic Shoulder Pain

**DOI:** 10.3389/fneur.2022.792695

**Published:** 2022-02-17

**Authors:** Xiaoya Wei, Guangxia Shi, Jianfeng Tu, Hang Zhou, Yanshan Duan, Chin Kai Lee, Xu Wang, Cunzhi Liu

**Affiliations:** ^1^International Acupuncture and Moxibustion Innovation Institute, School of Acupuncture-Moxibustion and Tuina, Beijing University of Chinese Medicine, Beijing, China; ^2^School of Life Sciences, Beijing University of Chinese Medicine, Beijing, China

**Keywords:** chronic shoulder pain, brain asymmetry, surface area, functional connectivity, precentral gyrus, postcentral gyrus

## Abstract

**Objective:**

The purpose of this study was to explore the structural and functional asymmetry of precentral and postcentral gyrus in patients with unilateral chronic shoulder pain (CSP) utilizing MRI.

**Patients and Methods:**

We collected structural and resting-state functional MRI (rs-fMRI) data in 22 left-sided, 15 patients with right-sided CSP, and 24 healthy controls (HCs). Here, we performed the structural asymmetry and seed-based functional connectivity (FC) analyses. We extracted regional cortical thickness and surface area measurements from T1-weighted MRI images, using asymmetry indexes (AIs) to assess asymmetries. We used Data Processing and Analysis for Brain Imaging software for seed-based FC analysis and selected unilateral-precentral and postcentral as the regions of interest. Then, we performed group comparisons of the neuroimaging metrics, and also explored the relationships between brain asymmetry and clinical variables.

**Results:**

We found significant differences in surface area AIs of the precentral among three groups, the AI values were negatively correlated with the visual analog scale score and positively correlated with Constant–Murley scores (CMS) in the left-sided CSP group. Further, FC of left postcentral with cingulate gyrus and left paracentral lobule showed significant group differences; FC of right postcentral with left caudate, left paracentral, and left postcentral were different among groups; FC of right precentral with the cingulate gyrus, precuneus, and left paracentral revealed significant group differences. Besides, there was a positive correlation between right precentral-cingulate gyrus FC and CMS in the right-sided CSP group.

**Conclusion:**

Surface area and FC patterns asymmetry exist in precentral and postcentral gyrus in patients with unilateral CSP. Asymmetry trend is associated with pain severity and shoulder joint function impairment. Brain structural and functional asymmetry may be an important indicator for understanding the potential mechanism of chronic pain.

## Introduction

In a variety of musculoskeletal disorders, chronic shoulder pain (CSP) is ranked third for the number of patients (1). Epidemiological surveys demonstrate that the lifetime prevalence rate of shoulder pain was ranging from 6.7 to 66.7% in the general population (2), and the incidence of chronic shoulder pain was 11% (1). Stiffness, limited motion, and persistent pain are common symptoms in patients (3). It is usually self-limited, resolving in 12–18 months among 40–50% of patients (4), yet the high recurrence rate and slow recovery impact their daily work and life. Due to the different etiologies, such as overload or strain, the affected sides of the shoulder could be different (5). A retrospective analysis revealed that compared with right-sided shoulder pain patients, the left-sided patients had higher scores on the Sickness Impact Profile (6).

According to the concept of homunculus (7), the cortical representation of motor and sensory functions of the shoulder are located in the precentral and postcentral, respectively. The primary motor area of the cerebral cortex is located in the precentral and has the functional characteristics of supporting the movement of the side limbs (8). The primary sensory cortex is located in postcentral, the characteristic of sensory projection is left and right cross (9). Accumulating evidence suggests that many chronic pain-related diseases, including CSP, showed extensive brain function and structural reorganization, such as the primary sensory cortex, primary motor cortex, paracentral, precuneus, cingulate cortex, and caudate (10–12). A study showed that the depth of sulcus in right precentral gyrus decreased in patients with CSP (10). As for patients with chronic neck and shoulder pain, the amplitude of the low-frequency fluctuations (ALFF) of their left precentral, right postcentral, left precuneus, and right cingulate were significantly reduced (11). These findings reflected that the precentral and postcentral gyrus are closely related to the neuropathological changes of CSP. However, these altered brain regions distributed in a wide range of the left or right hemispheres. We hypothesize that one of the most important factors may be the difference in affected sides of CSP.

The asymmetry of the structure and function of the two hemispheres is a distinctive feature of human brain (13). Hemispheric asymmetry changes are related to many mental and neurocognitive diseases, such as schizophrenia (14), autism (15), and obsessive-compulsive disorder (13). Moreover, changes in brain asymmetry have been found in chronic pain diseases. Recently, a chronic low-back pain imaging study (16) found interhemispheric asymmetry in the motor cortex, and cortical motor map of transversus abdominis and multifidus muscles is leftward asymmetric in 40.0% of participants. The asymmetry of the cortex in CSP remains unknown. Therefore, examining the lateralization effects in the cerebral cortex, especially in the precentral and postcentral gyri which are associated with the motor and sensory cortex, may be important for us to further understand the underlying mechanisms of CSP.

Here, this study focuses on assessing the structure and function asymmetry in precentral and postcentral regions in patients with unilateral CSP. We used the asymmetry index (AI) (17, 18) to assess the cerebral cortex thickness and the surface area of the precentral and postcentral regions. Seed-based FC analyses were adopted to evaluate FC patterns based on two pairs of symmetric seeds (left and right precentral and left and right postcentral). Then, we investigated whether left CSP (LCSP), right CSP (RCSP), and healthy controls (HCs) differed in their neuroimaging metrics. In addition, we investigated whether the structural and functional metrics were correlated with the clinical characteristics of pain symptoms.

## Methods

### Participants

In this study, 39 patients with unilateral CSP were recruited between December 2016 and July 2017. Moreover, we recruited 26 healthy controls, matching the age and gender of our patients (from March 2017 to December 2018). More details about the recruitment of the CSP and HC participants (19) were described in our previous papers. Briefly, the key inclusion criteria of CSP people were: (1) 45–65 years old; (2) right-handed; (3) shoulder pain duration from 6 weeks to 24 months; (4) visual analog scale (VAS) score between 50 and 100 mm. The key exclusion criteria were: (1) current therapy involving analgesia; (2) abnormal brain structures; and (3) other chronic pain conditions or a history of neuropsychiatric disorders.

This study was conducted in agreement with the Declaration of Helsinki. We collected MRI data from all participants. Before MRI scanning, VAS score (20–22) and Constant–Murley score (CMS) (23) were evaluated by participants. VAS (range 0–100 mm, 0 = no pain, 100 = worst pain) is a commonly used scale to evaluate the intensity of pain. CMS (range, 0–100 points) evaluates shoulder function of the patients. Higher CMS scores indicate better shoulder joint function and mobility.

### MRI Acquisition

MRI images were obtained at a Siemens 3.0 T MRI scanner (Skyra, Siemens, Erlangen, Germany) using a standard head coil at the Department of Radiology for Beijing Hospital of Traditional Chinese Medicine Affiliated to Capital Medical University. The high-resolution T1 MRI was acquired using gradient echo sequence with the following parameters: repetition time (TR) = 2,300 ms, echo time (TE) = 2.32 ms, flip angle (FA) = 8°, inversion time = 900 ms, field of view (FOV) = 240 × 240 mm, number of slices = 192, voxel size = 0.9375 × 0.9375 × 0.9 mm, and in-plane resolution = 256 × 256. In addition, the resting-state functional MRI (rs-fMRI) was scanned using echo planar imaging (EPI) sequence: TR = 2,000 ms, TE = 30 ms, FOV = 220 × 220 mm, FA = 90°, slice thickness/gap = 3.5/0.6 mm, axial slices = 33, in-plane resolution= 64 × 64, and 240 volumes. We used comfortable foam pads to minimize the head motion and earplugs to reduce noise interference. Before starting the scanning, we instructed participants to keep their eyes closed, stay awake, avoid engaging in any specific thoughts, and keep still.

### Image Processing

Using the “recon-all” command implemented in FreeSurfer (http://surfer.nmr.mgh.harvard.edu/, V6.0) to process the structural MRI data to reconstruct the cortical surface. Mean cortical thickness and surface area were derived for each of the 68 cortical regions of the Desikan-Killiany Atlas (34 per hemisphere). Cortical thickness was estimated for each participant using the distance from the white matter boundary to the corresponding pial surface (24). Cerebral surface area was calculated by mesh generation and surface triangulation. The cortex thickness and surface area of each hemisphere are performed independently.

Furthermore, functional MRI (fMRI) data were processed using the software MATLAB 2013b (MathWorks, Natick, MA, USA) and the toolkit of Data Processing and Analysis for Brain Imaging (DPABI version 5.1, http://www.rfmri.org/dpabi) (25). For each image data of participant, we discarded the first 10 volumes because of signal equilibrium, a total of 230 volumes for each subject were processed with the slice timing, motion correction, spatial smoothing (6-mm FWHM), and spatial normalization to the Montreal Neurological Institute (MNI) space. Then, we re-sampled the data into 3 × 3 × 3 mm^3^. Finally, after removing the linear trend, we applied a 0.01–0.08 Hz bandpass filter.

### Quality Control

Four participants (two patients and 2 HCs) were excluded from the study on account of excessive head motion (>3 mm in translation or >3.0° in rotation) during the rs-fMRI scanning. As a result, 22 patients with LCSP, 15 patients with RCSP, and 24 HCs were included in further statistical analyses. Furthermore, we extracted the mean framewise displacement (FD) for each participant to measure the extent of head motion (26, 27) and compared them among the three groups. The non-parametric test result showed that there is no significant difference in head motion among the three groups (H = 4.137, *p* = 0.126).

### Clinical and MRI Statistical Analyses

#### Demographic and Clinical Characteristics Analyses

We used SPSS statistics 21 software to conduct statistical analyses. Before statistical analyses, we checked the normality of each metric. Age and CMS were normally distributed in each group, whereas pain degree (VAS score) and pain duration were non-normally distributed. We used one-way ANOVA for age, two-sample *t*-test for CMS, and Mann–Whitney *U*-non-parametric tests for VAS and pain duration. As for categorical variables (i.e., gender), we used the chi-square test to evaluate the differences among groups.

#### Surface-Based Morphometry Analyses

To compare the difference in cortex structural asymmetry of precentral and postcentral regions related to unilateral CSP, AI for each cortical metric is calculated as a widely used formula (28):


$$\begin{array}{l} AI=\frac{\left(Left-Right\right)}{\left(Left+Right\right)/2} \end{array}$$

Consequently, a negative AI reflects a rightward asymmetry and a positive AI means that asymmetry is shifted to the left. In the three groups, we used ANOVA or non-parametric tests to compare the difference in asymmetrical changes in cortical surface area and cortical thickness, and then performed the *post-hoc* analysis.

#### Seed-Based Functional Connectivity Analyses

For the functional analyses, we selected left precentral, right precentral, left postcentral, and right postcentral from the Automated Anatomical Labeling (AAL) atlas (29) as the seeds because AAL is a commonly used atlas in functional space (30–34). We performed Pearson's correlation between each seed region and the whole brain voxel. Then, Fisher's *r*-to-*z* transformation was used to convert each final FC map of individual to *z*-value maps. Among three groups, we used one-way ANOVA analyses to obtain the *F* statistical map. Finally, Gaussian Random Field (GRF) theory multiple comparison correction (35) was used for all maps (voxel-level *p* < 0.01 and cluster-level *p* < 0.05).

#### Brain Alteration and Clinical Variables Analyses

We extracted FC value between the seed and the region with significant group differences. For normally distributed variables, Pearson's correlation was used to analyze the correlation between AI and FC values and VAS and CMS. For non-normally-distributed variables, we used Spearman's correlation analysis. The above statistical analysis used SPSS software (significance level is *p* < 0.05).

## Results

### Participants

Among the three groups, there was no significant difference here in demographic data (age, *F* = 0.714, *p* = 0.494; gender, *X*^2^ = 3.795, *p* = 0.150). The pain duration (*Z* = 0.422, *p* = 0.680), VAS (*Z* = 0.363, *p* = 0.725), and CMS (*t* = 0.834, *p* = 0.410) showed no significant differences in patients with LCSP and RCSP. More details are presented in Table 1.

**Table 1:** Demographic and clinical characteristics of patients with chronic shoulder pain (CSP) and healthy control (HC).

**Parameter**	**LCSP patients (*n* = 22)**	**RCSP patients (*n* = 15)**	**HC (*n* = 24)**	**Statistics**	***P*-value**
Age (year)^#^	54.18 ± 6.03	54.20 ± 4.81	55.83 ± 4.72	*F* = 0.714^a^	0.494
Gender (Male/Female)	11/11	11/4	10/14	*X*^2^ = 3.795^b^	0.150
Pain duration (months)^*^	4 (2–24)	5 (1–24)	N/A	*Z* = 0.422^c^	0.680
Pain degree (VAS)^*^	65 (50–100)	60 (55–100)	N/A	*Z* = 0.363^c^	0.725
Shoulder function (CMS)^#^	54.55 ± 13.29	50.47 ± 16.38	N/A	*t* = 0.834^d^	0.410

*For continuous variables, we used mean (SD)*

# *if the measurements were normally distributed, and median (minimum to maximum)*

* *if the measurements were not normally distributed*.

a *One-way ANOVA*.

b *Chi-square test*.

c *Non-parametric test: Mann–Whitney U*.

d *Two-sample t-test*.

*N/A, not applicable; VAS, visual analog scale score, 0–100 mm; CMS, the Constant–Murley score, 0–100 points; LCSP, left chronic shoulder pain; RCSP, right chronic shoulder pain; HC, healthy controls*.

### Brain Structural Asymmetry

**Figure 1:**
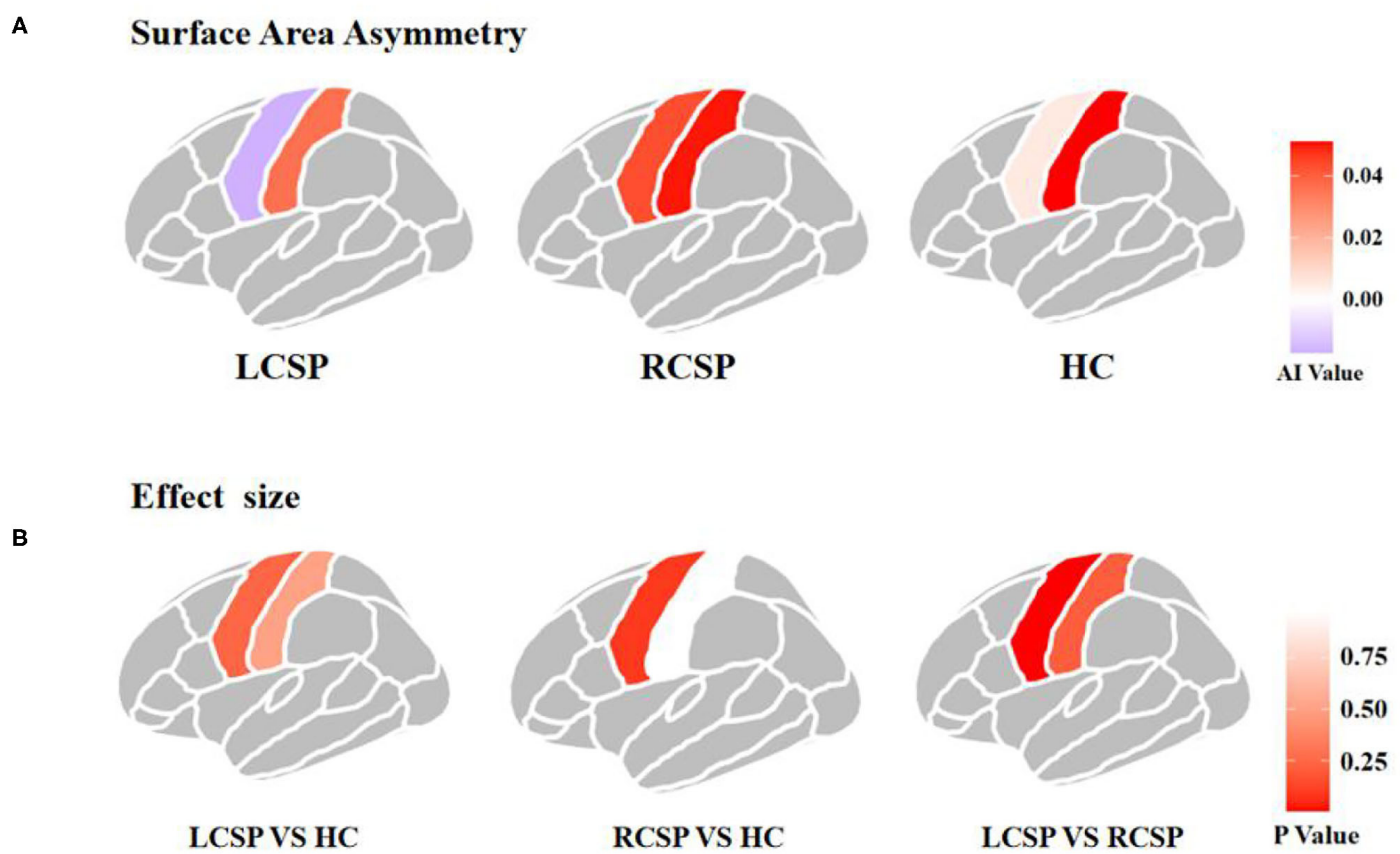
Asymmetry of surface area and effect sizes in the precentral and postcentral gyrus in three groups. **(A)** Asymmetry of surface area in the precentral and postcentral gyrus. Colors indicate the directions of average interhemispheric differences, with red indicating leftward asymmetry, and purple indicating rightward asymmetry. **(B)** Region-wise effect sizes of hemispheric asymmetry. Effect sizes are Cohen's *d* values.

Comparisons among the three groups using ANOVA and non-parametric testing revealed that the precentral region had significantly different brain asymmetry in surface area (*F* = 3.958, *p* = 0.024). According to the *post-hoc* pairwise comparison analyses, there was a significant difference in AI between the LCSP and RCSP groups (ES = −1.002, *p* = 0.005), specifically, the LCSP group showed rightward asymmetries (group means AI = −0.017), and the RCSP group showed leftward asymmetries (group means AI = 0.043) in the precentral surface area (Table 2; Figure 1). However, we did not find a significant difference in AI of cortical thickness in both the precentral and postcentral regions (Supplementary Material).

**Table 2:** Differences in surface area asymmetry index among left CSP (LCSP), right CSP (RCSP), and HC.

**Region**	**LCSP patients**	**RCSP patients**	**HC**	**Statistics**	***P*-value**	***Post-hoc*** **pairwise comparison**					
	***N* = 22**	***N* = 15**	***N* = 24**	***X*^2^/*F***		**LCSP vs. HC**	***P*-value**	**RCSP vs. HC**	***P*-value**	**LCSP vs. RCSP**	***P*-value**
Postcentral	0.036 (−0.201~0.122)^$^	0.050 ± 0.064	0.051 ± 0.098	*X*^2^ = 1.461^a^	0.482	*Z* = 0.660^c^	0.509	−0.012^#^	0.972	*Z* = −1.268^c^	0.213
Precentral	−0.017 ± 0.061	0.043 ± 0.057	0.006 ± 0.068	*F* = 3.958^b^	0.024^*^	−0.348^#^	0.245	0.572^#^	0.091	−1.002^#^	0.005^*^

*We used mean (SD) if the measurements were normally distributed, and the median (minimum to maximum)*

$ *if the measurements were not normally distributed*.

* *Survives Bonferroni correction*.

a *Kruskal–Wallis test*.

b *One-way ANOVA*.

c *Non-parametric test: Mann–Whitney U*.

# *Cohen's d of two-sample t-test*.

*LCSP, left chronic shoulder pain; RCSP, right chronic shoulder pain; HC, healthy controls. The bold values indicate the significant results (p < 0.05)*.

### Brain Functional Asymmetry

**Figure 2:**
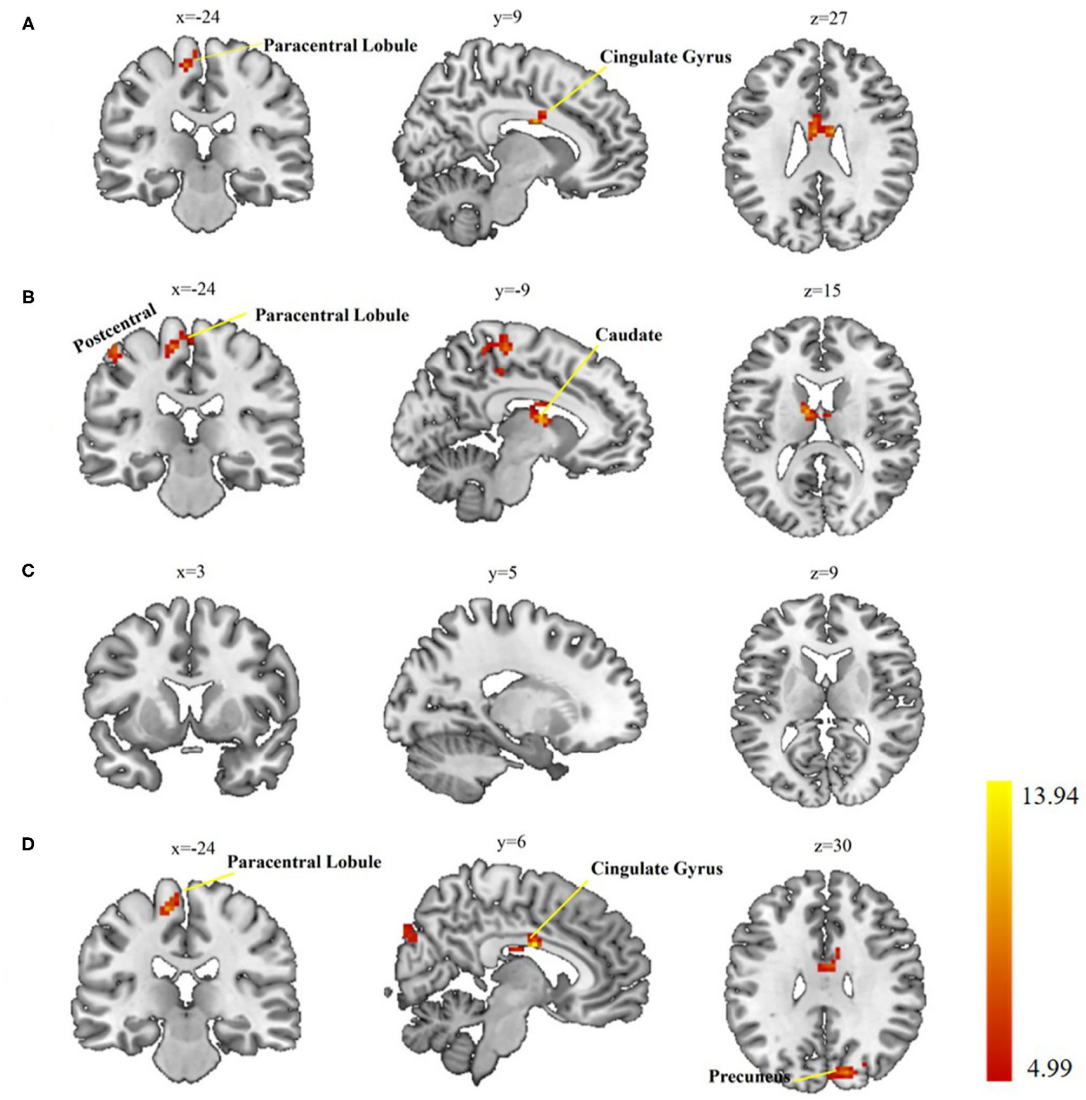
Group differences in seed-based functional connectivity (FC). **(A)** Significant regions based on left postcentral seed; **(B)** significant regions based on right postcentral seed; **(C)** significant regions based left precentral seed; **(D)** significant regions based on right precentral seed. Gaussian Random Field (GRF) theory correction with voxel level *p* < 0.01 and cluster level *p* < 0.05.

When comparing the left postcentral seed-based FC maps among the three groups, two brain regions with significant differences were found, cingulate gyrus and paracentral lobule (voxel-level *p* < 0.01, cluster-level *p* < 0.05, cluster size > 55 voxels; Table 3, Figure 2A). Otherwise, the caudate, paracentral lobule, and postcentral revealed different FC with the right postcentral seed (voxel-level *p* < 0.01, cluster-level *p* < 0.05, cluster size > 51 voxels; Table 3, Figure 2B). However, no brain regions showed a significant FC difference with the left precentral seed (Figure 2C). For the right precentral seed, FC maps were significantly different in the cingulate gyrus, precuneus, and paracentral lobule (voxel-level *p* < 0.01, clusterlevel *p* < 0.05, cluster size > 47 voxels; Table 3, Figure 2D).

**Table 3:** Differences in seed-based functional connectivity (FC) among LCSP, RCSP, and HC.

**Seed**	**Connectivity regions**	**Peak MNI coordinates**			**Voxels size**	***F*-value**
		**X**	**Y**	**Z**		
Left postcentral	Cingulate gyrus	9	−3	27	55	10.769
	Left paracentral	−12	−24	63	61	9.264
Right postcentral	Left caudate	−9	−3	15	66	11.093
	Left paracentral	−12	−24	63	99	9.639
	Left postcentral	−48	−27	63	59	9.535
Left precentral	–	–	–	–	–	–
Right precentral	Cingulate gyrus	6	−3	27	102	13.940
	Precuneus	15	−81	30	64	9.876
	Left paracentral	−12	−24	63	61	10.644

*MNI, montreal neurological institute; GRF, gaussian random field theory, Voxel p < 0.01, cluster p < 0.05*.

To rule out the possibility that our results depended on the choice of the atlas, we re-performed seed-based FC analyses using the precentral and postcentral seeds taken from the Desikan-Killiany atlas. We extracted the value of the eight significant regions (Table 3) from the FC maps of individuals' based on the Desikan-Killiany atlas and found that the FCs were still significantly different among the three groups (Table 4).

**Table 4:** Differences in Desikan-Killiany-seed-based FC among LCSP, RCSP, and HC.

**Groups-FC map**	**Seed**	**Peak MNI coordinates**			***F*-value**	***P***
		**X**	**Y**	**Z**		
Left postcentral	Cingulate gyrus	9	−3	27	12.497	<0.001^**^
	Left paracentral	−12	−24	63	8.396	<0.001^**^
Right postcentral	Left caudate	−9	−3	15	3.227	0.047^*^
	Left paracentral	−12	−24	63	9.682	<0.001^**^
	Left postcentral	−48	−27	63	14.075	<0.001^**^
Left precentral	–	–	–	–	–	–
Right precentral	Cingulate gyrus	6	−3	27	10.894	<0.001^**^
	Precuneus	15	−81	30	9.197	<0.001^**^
	Left paracentral	−12	−24	63	11.746	<0.001^**^

* *p < 0.05*.

** *p < 0.001*.

### Correlation Analysis Results Between AI, FC, and VAS, CMS

**Figure 3:**
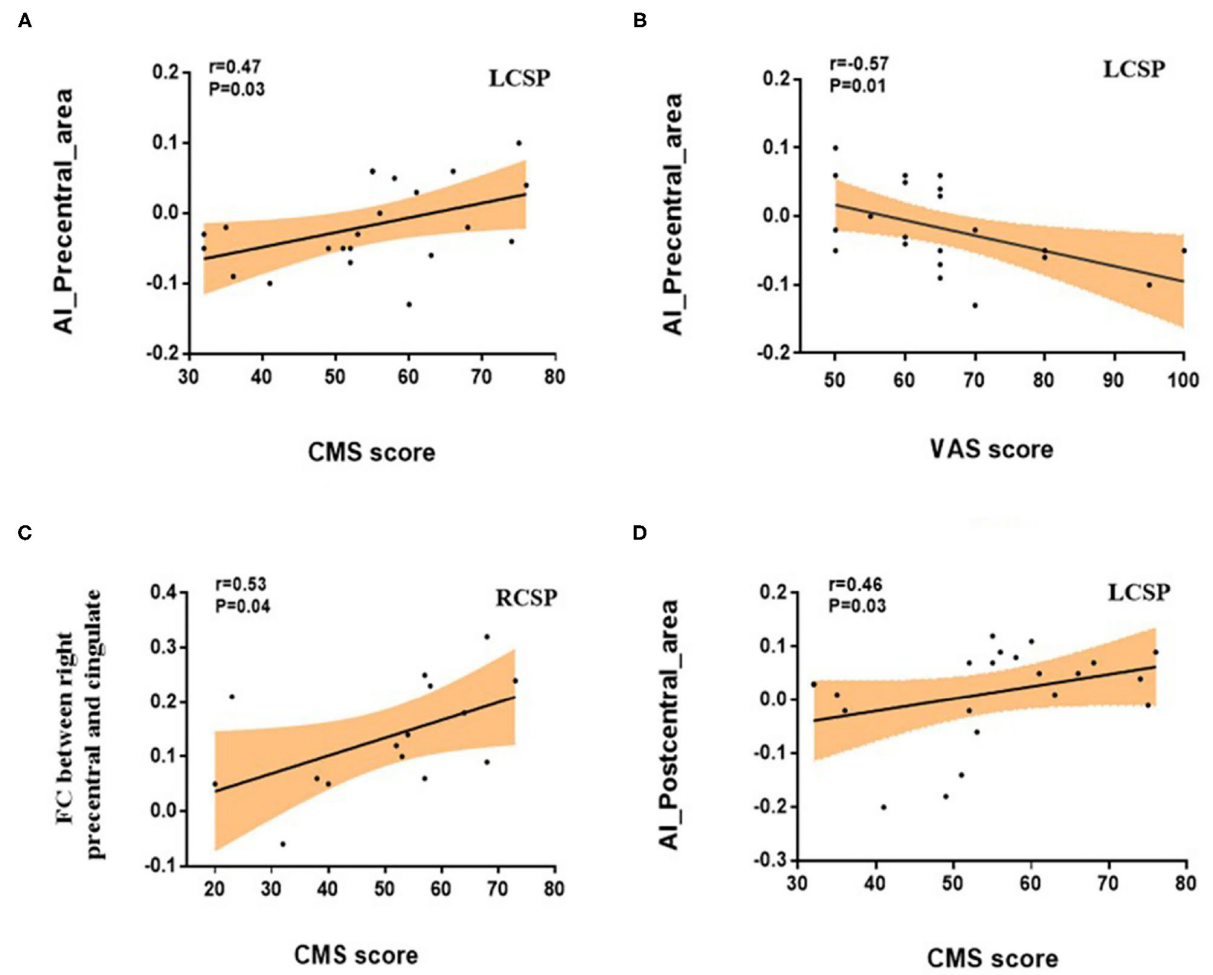
Correlations between brain index and clinical variables in the patients with chronic shoulder pain (CSP). **(A)** Pearson's correlation showed a positive association between the asymmetry index (AI) of precentral surface area and the CMS scores (*r* = 0.47, *p* = 0.03). **(B)** Spearman's correlation showed a negative association between the AI of precentral surface area and the VAS scores (*r* = −0.57 *p* = 0.01). **(C)** Pearson's correlation showed that FC between right precentral gyrus and cingulate gyrus was significantly positively correlated with CMS (*r* = 0.53, *p* = 0.04). **(D)** Spearman's correlation showed a positive association between the AI of postcentral surface area and the CMS scores (*r* = 0.46 *p* = 0.03).

Correlation analyses revealed that the AI values of the precentral surface area had a positive correlation with the CMS scores (Pearson's *r* = 0.47, *p* = 0.03) and a negative correlation with the VAS scores (Spearman's *r* = −0.57, *p* = 0.01) in the patients with LCSP (Figures 3A,B). In addition, there was a significant positive correlation between FC and CMS between the right precentral gyrus and cingulate gyrus (*r* = 0.53, *p* = 0.04) in the RCSP group (Figure 3C). Although there was no difference in the cortical area of the postcentral region between the three groups, we still found a positive association between AI of the postcentral surface area and CMS scores (Spearman's *r* = 0.46, *p* = 0.03) in patients with LCSP patients (Figure 3D).

## Discussion

Based on our hypothesis, we evaluated the structural and functional asymmetry of precentral and postcentral gyrus in patients with unilateral CSP. For the cortical structural asymmetry analysis, we found significant differences in the surface area of precentral among the left-sided CSP, right-sided CSP, and HC groups. Seed-based FC analyses revealed different connectivity patterns in patients with CSP for two pairs of symmetric seeds (left vs. right precentral, left vs. right postcentral). In addition, we found these structural and functional asymmetry metrics were correlated with clinical parameters. Specifically, AI values of precentral in LCSP had a significant positive correlation with CMS scores and a negative correlation with the VAS scores. Right precentral—cingulate gyrus FC had a significant positive correlation with CMS scores in RCSP. Our findings may clarify the neural mechanism of CSP from the perspective of brain asymmetry.

### Surface Area Asymmetry of the Precentral Gyrus

A previous meta-analysis (36) has shown that the right precentral gyrus undergoes structural changes in some chronic pain diseases, such as chronic back pain, migraine, or chronic facial pain. Compared with HC, patients with chronic neck pain (CNP) had a smaller cortex in right precentral area, and the study has shown a correlation between the volume changes of the right precentral and degree of neuromuscular control (37). However, these studies only found the chronic pain-related structural changes in the unilateral (i.e., right) precentral gyrus and ignored the possible brain asymmetry substrates. Besides, the affected sides of chronic pain were not considered separately. Interestingly, ANOVA comparing the three groups in the present study showed a significant effect of group, but the *post-hoc* difference was observed only between RCSP and LCSP groups and not between HC and patient groups. Here, the AI of the precentral area had a tendency of rightward asymmetry in the LCSP group, and a tendency of leftward in the RCSP group. The AI of HC was in between. These findings indicate that the precentral areas of the contralateral and ipsilateral to the shoulder pain side are affected asymmetrically. This is consistent with the left-right cross characteristic of sensory/motional projection that the precentral may be more involved to the pain and function of the contralateral shoulder. Our results may further support the lateralization of the brain in pain processing (38–40).

In addition to the precentral gyrus, accumulating evidence has demonstrated that the motor cortical reorganization and asymmetries are associated with many chronic pain diseases (16, 41–43). It was reported that the cortical motor map of 40.0% chronic low-back pain (CLBP) group subjects was leftward asymmetric compared with HC (16). Phantom limb pain (43) patient's motor cortex reorganizes asymmetrically, and the volume of gray matter in the affected hemisphere is reduced. Further studies are needed to investigated the whole motor cortex and reveal the neural asymmetry basis of the CSP or other chronic pain diseases.

Moreover, we found associations of the precentral area's AI with VAS and CMS in LCSP group, indicating that the lower rightward structural asymmetry of precentral may relate to the lower pain degree and better shoulder function (44). However, the VAS and CMS were not correlated with the AI values of the precentral area in RCSP group. We speculate that the difference between the groups could be accounted by different etiologies because the gender proportions and samples in each group were not identical. Particularly, there were only 4 women and 15 sample size in RCSP group. Besides, different potential mechanisms related to handedness may also lead to the observation of such differences. All the participants were right-handed in the present study. Previous study has shown that right-handed patients with left-sided pain had poorer physical functioning than right-sided pain patients (6). Further studies with left-handed participants will be helpful to test this speculation.

### FC Pattern Asymmetry of the Precentral and Postcentral Gyrus

Regarding the FC of the right precentral, we found that the connection with the precuneus, cingulate, and left paracentral lobule increased. Our results support previous research, these brain areas play an important role in chronic pain-related diseases (37, 45–47). The cingulate gyrus is related to various cognitive, social, and emotional functioning (45). We speculate that the emotional function of patients with chronic shoulder pain will be affected. However, we cannot confirm this view because the emotional/cognitive ability of patients was not measured in this study. The precuneus is part of a group of areas related to the neurological characteristics of pain (36) and acts as an antinociceptive region (37) and increased FC in the precentral gyrus with the superior parietal cortex in patients with CNP (47). However, in present results, no brain areas with FC were reported with left precentral, it is reasonable to speculate that there may also exist functional asymmetry in the precentral gyrus in patients with CSP.

In this study, four seeds were selected according to the AAL atlas to better disclosure the FC patterns of the different hemispherical precentral and postcentral. Specifically, the paracentral lobule showed increased FC from all three seed points. The paracentral lobule that plays a pivotal role in the location and identification of pain (48) with the postcentral, which is located in the upper medial part of the precentral. It has been reported that the excitability of the motor cortex in patients with chronic pain has changed, such as paracentral lobules, which may be affected by the underlying pathological properties of different diseases (49, 50). For example, in a CLBP study, increased ALFF in the bilateral postcentral, precentral, and paracentral were found in patients (51). In our study, we found only enhanced functional connectivity of the left paracentral lobule with each seed and our findings may support the pivotal role of paracentral lobules in pain regulation of CSP.

The caudate nucleus is an important part of the basal ganglia. It is involved in the reward circuit in chronic pain and involves the motivational and emotional aspects of behavior, such as rewards, which are important for planning and decision-making (52). Many previous studies have reported that the caudate nucleus has hemispheric asymmetry (53–55). For instance, In a KOA imaging study, it was found that the volume of the caudate nucleus was rightward hemispheric asymmetry (56). The GRF-corrected FC analysis showed that only the FC between the right postcentral and left caudate nucleus had significant group differences. Moreover, decreased bilateral caudate nucleus coactivation has been found in patients with multiple sclerosis (MS) (57). Instead, we found enhanced FC between the right postcentral gyrus and the caudate nucleus. The reason for the difference may be caused by the different pathogenesis and different clinical characteristics of MS and CSP. Our findings add new evidence of brain asymmetry to previously published findings in CSP patients of brain alterations.

Our study has some limitations. First, the sample size of this study is not very large, and there are only four female patients in the RCSP group. Hence, the conclusion needs to be repeated in a large unilateral CSP population having equal numbers of men and women. Second, our research is essentially a cross-sectional study. In the future, we will conduct longitudinal studies with other chronic pain-related diseases, exploring whether the brain structural and functional asymmetry will change after treatment. Third, this study focused only on the impact of lateralization on the two regions. In the future, we will further explore the differences in another brain anatomy (e.g., white matter architecture and gray matter volume), and altered functioning between networks in patients.

## Conclusion

In summary, our study found that there was significant asymmetry in the cortical surface area of the precentral in patients with CSP, and the asymmetry value of patients with LCSP has a clear correlation with the severity of the condition of patient. Additionally, the precentral and postcentral gyrus in different hemispheres had different FC patterns with the whole brain regions in patients with CSP. What's more, the FC between the right precentral and cingulate gyrus was correlated with the condition of patient in the RCSP group. Our research adds to the literature suggesting a critical role of precentral and postcentral in the pathophysiology of CSP, and the brain asymmetry effect may be an important hallmark of chronic pain diseases.

## Data Availability Statement

The raw data supporting the conclusions of this article will be made available by the authors, without undue reservation.

## Ethics Statement

The studies involving human participants were reviewed and approved by the Ethical Committee of the Beijing Hospital of Traditional Chinese Medicine, affiliated with Capital Medical University. The patients/participants provided their written informed consent to participate in this study.

## Author Contributions

XW designed this work. XYW, HZ, and JT analyzed the data. XYW and GS discussed the results. XYW wrote the initial draft. XYW, XW, GS, YD, CL, and CKL substantially revised the manuscript. All authors agreed to be accountable for all aspects of this work.

## Funding

This work was supported by the Scientific Research Foundation for high-level talents of Beijing University of Chinese Medicine and the Beijing Technology Development of Traditional Chinese Medicine Foundation–Annual Planning Project (ref: JJ 2013-40).

## Conflict of Interest

The authors declare that the research was conducted in the absence of any commercial or financial relationships that could be construed as a potential conflict of interest.

## Publisher's Note

All claims expressed in this article are solely those of the authors and do not necessarily represent those of their affiliated organizations, or those of the publisher, the editors and the reviewers. Any product that may be evaluated in this article, or claim that may be made by its manufacturer, is not guaranteed or endorsed by the publisher.
