# Supplementary material for: Structural and Functional Asymmetry in Precentral and Postcentral Gyrus in Patients With Unilateral Chronic Shoulder Pain
Source: Front Neurol. 2022 Feb 17;13:792695. doi: 10.3389/fneur.2022.792695 (PMC8892006; doi:10.3389/fneur.2022.792695)
Supplement: Supplementary file 1 [file Table_1.DOCX]

**supplementary material**

| **Supplement 1 Difference in cortical thickness asymmetry index among LCSP, RCSP and HCs** | | | | | |  |
| --- | --- | --- | --- | --- | --- | --- |
| Region | LCSP Patients N=22 | RCSP Patients N=15 | HCs N=24 | Statistics | P value |  |
|  |  |  |  |  |  |  |
| postcentral | 0.010±0.023 | 0.004±0.022 | -0.002±0.036 | H=1.394 | 0.498 |  |
| precentral | 0.004±0.024 | 0.023±0.030 | 0.005±0.031 | F=2.528 | 0.089 |  |
